# Un hémothorax gauche révélant une dissection de l`aorte: à propos d'un cas

**DOI:** 10.11604/pamj.2014.18.235.1338

**Published:** 2014-07-22

**Authors:** Mouhamadou Nazirou Doddo Siddo, Malick Bodian, Falikou Diaby, Soulemane Pessinaba, Mouhamadou Bamba Ndiaye, Maboury Diao, Alassane Mbaye, Simon Antoine Sarr, Sarah Mouna Coly, Adama Kane, Moustapha Sarr, Serigne Abdou Ba

**Affiliations:** 1 Service de cardiologie, CHU Aristide Le Dantec, Dakar, Sénégal; 2 Service de cardiologie, Hôpital régional de Zinguichor, Sénégal; 3 Service de cardiologie, Hôpital Général Grand Yoff, Dakar, Sénégal

**Keywords:** Dissection, aorte, hémothorax, Dissection, aorta, hemothorax

## Abstract

Certaines dissections de l`aorte peuvent se compliquer d'hémothorax faisant, évoquer initialement, une pathologie pleuro-pulmomaire. Nous rapportons l'observation d'un patient âgé de 67 ans, hypertendu, atteint d`une dissection de l`aorte, présentant un tableau typique, clinique et radiologique d`un syndrome d`épanchement pleural gauche en dehors de tout contexte infectieux et traumatique. Le tableau d`un syndrome pleural gauche a conduit à la réalisation d`une ponction pleurale qui a ramené un liquide hémorragique. La tomodensitométrie thoracique a conclu à une dissection aortique type B de Stanford. La prise en charge chirurgicale est indiquée en cas de complication comme ce fut le cas de notre patient.

## Introduction

La dissection aortique est une affection très grave souvent mortelle et qui est révélée par des symptômes variés. L'affection se révèle rarement par un hémothorax. Nous rapportons un cas d'hémothorax gauche révélant une dissection de l'aorte descendante.

## Patient et observation

**Figure 1:**
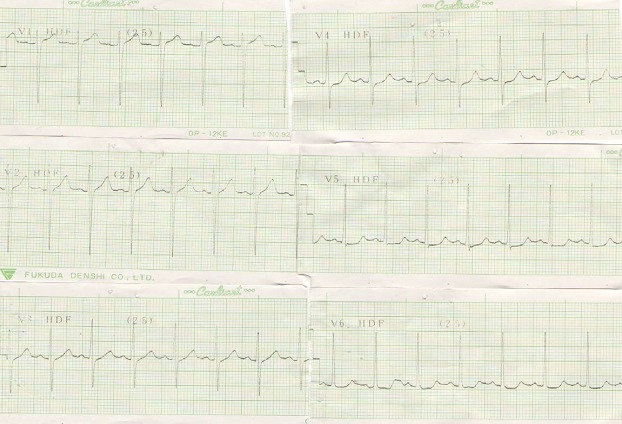
Electrocardigramme

**Figure 2:**
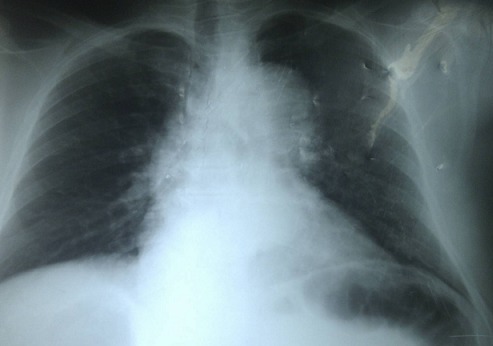
Radiographie du thorax avant admission en cardiologie

Mr A.C., âgé de 67 ans, suivi pour hypertension artérielle de façon irrégulière depuis 2 ans sous Adalate^®^ (Nifédipine 20 mg par jour) était admis le 27 mars 2011 dans le service de cardiologie de l'Hôpital régional de Ziguinchor pour une symptomatologie faite d'une douleur basithoracique gauche. Celle-ci d'apparition brutale évoluait depuis quatre jours, intense, permanente, à type de constriction, sans irradiation particulière. L'interrogatoire n'a pas retrouvé de notion de traumatisme ni de contexte infectieux. Cette symptomatologie avait motivé une consultation avec une exploration paraclinique dont un électrocardiogramme (Figure 1) et un téléthorax (Figure 2). Devant la persistance de la douleur sous antalgique (Tramadol) et l'apparition d'une dyspnée, le patient fut admis au service de Cardiologie de l'Hôpital Régional de Ziguinchor pour une meilleure prise en charge.

L'examen clinique a mis en évidence, une pression artérielle à 100/80 mmHg aux deux bras, une température à 36,8°C et une fréquence cardiaque à 110 battements par minute. On notait une obésité mixte modérée avec une taille à 1,72 m, un poids à 96 kg pour un IMC à 32,54kg/m^2^ et un tour de taille = 106 cm. Un syndrome d’épanchement pleural liquidien occupant la quasi-totalité du poumon gauche était retrouvé. Le reste de l'examen somatique était banal.

La CRP était élevée à 48 mg/L et la vitesse de sédimentation accélérée à 60 mm à la première heure. La numération formule sanguine a mis en évidence une hyperleucocytose à 19.400 éléments par mm^3^, une anémie normochrome, normocytaire avec une hémoglobine à 9,0 g/dl. Le reste du bilan biologique était normal.

**Figure 3:**
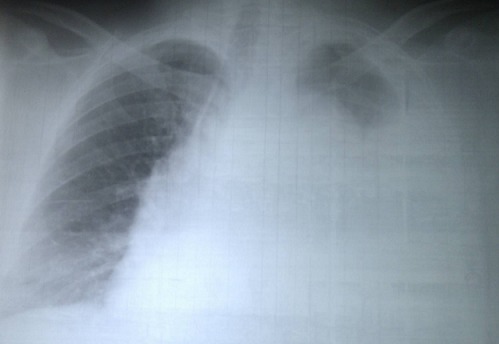
Radiographie du thorax à l'admission en cardiologie

La radiographie thoracique révélait une opacité pleurale, occupant la quasi-totalité du champ pulmonaire gauche, avec un élargissement du médiastin (figure 3). L’échocardiographie Doppler n`a pas pu être réalisé devant un patient instable Une ponction pleurale exploratrice réalisée, a ramené un liquide hémorragique, avec un taux d'hématocrite > 50%.

**Figure 4:**
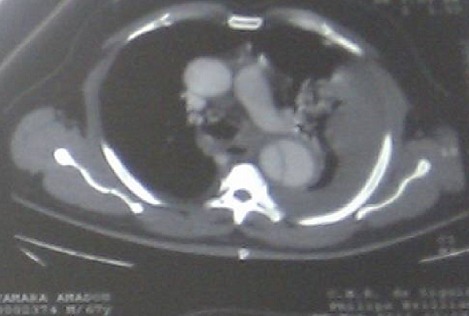
Angioscanner thoracique

Les anomalies radiologiques et le résultat du liquide de ponction pleurale avaient fait évoquer le diagnostic de dissection aortique compliquée de rupture dans la grande cavité pleurale gauche et justifiant donc, la réalisation d`une tomodensitométrie thoracique qui a mis en évidence un flap intimal avec présence de deux chenaux étendus de la crosse de l`aorte jusqu`à l`aorte abdominale en rapport à une dissection de type B de Stanford (type III de De Bakey), compliquée d'une fissuration et d'un épanchement pleural gauche (figure 4). Le patient a bénéficié d'un traitement antalgique et antihypertenseur contenant un bêtabloquant (Aténolol 100 mg par jour) ayant permis de contrôler sa pression artérielle. L’évolution a été défavorable et le décès est survenu 48 heures après le diagnostic dans un tableau de collapsus cardio-vasculaire.

## Discussion

Les hémothorax sont, dans la majorité des cas, secondaires à un traumatisme, à une technique invasive thoracique ou à certaines pathologies pulmonaires ou pleurales. Un hémothorax secondaire à une dissection aortique est rare [1]. Il fait généralement suite à une rupture de l'aorte thoracique descendante, initialement dans la région médiastinale, puis dans l'espace pleural gauche en raison de la proximité de la cavité pleurale [2], comme cela est le cas de notre patient. Sa survenue semble beaucoup plus rare, estimée à environ 10% des cas de dissection aortique [3].

Hara et al [4] dans une étude portant sur 48 patients ayant une dissection de l`aorte, ont rapporté seulement 3 cas d'hémothorax. Sur le plan clinique, la douleur thoracique est le principal symptôme, mais environ 5% des patients sont asymptomatiques, essentiellement les sujets âgés [5]. Les facteurs péjoratifs sont: l’âge avancé, l'hypertension artérielle diastolique, et la maladie artériosclérotique disséminée [5].

La tomodensitométrie avec injection de produit de contraste est actuellement la méthode de choix pour le diagnostic. En plus d'une aorte dilatée, elle permet de voir le faux chenal et le «flap» intimal, mais aussi de montrer un éventuel épanchement pleural ou péricardique associé [4].

Notre patient présente une DA type III de De Bakey et type B de Stanford qui représente 40% des DA. Le traitement dépend du siège de la DA: la dissection de l'aorte descendante non compliquée (type B de Stanford) doit être traitée médicalement par un contrôle strict de la tension artérielle. Le traitement chirurgical doit être indiqué en urgence, pour les DA de type A, alors que pour le type B l'intervention n'est justifiée qu'en cas de complication comme dans le cas de notre patient. L’évolution dépend du mode de rupture et de la capacité des structures avoisinantes, particulièrement la plèvre médiastinale, à contenir l'hémorragie [1].

## Conclusion

Le diagnostic de dissection aortique doit être évoquée devant tout hémothorax inexpliqué surtout lorsqu'il s'agit d'un sujet âgé, hypertendu et présentant une anomalie de l'arc aortique à la radiographie du thorax.
